# Supplementary material for: Consumer attitudes and perceptions on consumption of edible insects among communities in western Kenya
Source: PLoS One. 2025 Feb 25;20(2):e0318711. doi: 10.1371/journal.pone.0318711 (PMC11856288; doi:10.1371/journal.pone.0318711)
Supplement: Attachment — Submitted filename: Response to Reviewers.doc [file pone.0318711.s002.doc]

| **Editor** | The authors should submit the following data behind the means, standard deviations and other measures reported; | The authors have submitted all the raw data required to replicate the results of your study | |
| --- | --- | --- | --- |
| **Reviewer #1:** | | | |
| **Section** | **Reviewer comments** | **Author response** | **Document page and line number** |
| Title | The title and abstract clearly and concisely indicate the focus of the manuscript | Thank you for your comment. |  |
| Data analysis | It is essential to ensure that all text is consistent in color, as this can be identified in the software reference. | Thank you for the correction. We have harmonized the text color. | Page 3; line 119 |
| Results | I suggest that examples of participants' statements be presented in the same format as in Example 3.2, with proper indentation, spacing, and enclosed in quotation marks. | Thank you for the suggestion. We have harmonized the format as suggested. | Page 6; Lines 189-193 |
|  | How were the insects consumed? Were their forms of preparation or presentation described (e.g., dried, fresh, crushed)? Providing this detail would add clarity and context to the study. | Thank you for this important comment. We have included information on forms of preparation. | Page 4; Line 141-142  Page 8; Lines 256-258 |
| Discussions | I recommend organizing the discussion in a structured manner, following the approach used in the introduction and methods. | Thank you for this important comment. We have reorganized the discussion section. | Pages 10-11; lines 312, 318, 329 |
|  | Consider discussing the risks to food and nutritional security associated with encouraging this production and consumption. Emphasize the potential health risks involved and propose measures to enable users to produce and consume these products safely, avoiding contamination during collection, market availability, and general consumption. | Thank you for this important comment. We have included information on health risks involved. | Page 11; lines 353-356 |
|  | I suggest including additional references to support the data presented in the second paragraph of this section. | Thank you for this important comment. We have included additional references to support our discussion. | Page 11; Lines 326-343 |
|  | Furthermore, I recommend incorporating future research perspectives along with the study's limitations in a separate section following the discussion. | Thank you for this important comment. We have included information on study limitations and future research perspectives. | Page 12-13; lines 400-407 |
| Conclusion | Alongside summarizing the main findings, it is important to outline prospects for future research and provide an overall conclusion of the study, while also detailing the results individually. | Thank you for this important comment. We have revised the conclusions to include future research prospects and an overall conclusion. | Page 13; Lines 398-416 |
| References | I recommend including more recent references, as it was observed that 60% of the references are over five years old | Thank you for this important comment. We have included recent references in the introduction and discussion sections. | Page 1-2; Lines 45-67  Page 11-12; Lines 309-386’ All the new references have been listed and highlighted in yellow |

| **Reviewer #2:** | | |
| --- | --- | --- |
| **Reviewer comments** | **Author response** | **Document page and line number** |
| The paper is limited to FGDs in only two countries, Kisumu and Vihiga. While informative, this narrow geographical focus may not capture the diversity of attitudes across Kenya or even other regions within western Kenya. A more comprehensive study with broader regional representation would enhance the generalizability of the findings. | Thank you for this important comment. This was a pilot study that focused on providing preliminary findings to inform a larger future study to capture diverse attitudes within the broader population. This is recognized as a study limitation. | Page 11; line 354-361 |
| While the study involved 59 respondents, the demographic representation might not fully capture the diverse attitudes within the broader population. Increasing the sample size and ensuring a wider range of ages, education levels, and socio-economic backgrounds could enhance the robustness of the conclusions drawn. | Thank you for this important comment. The focus of this study was to provide preliminary findings to inform a larger future study to capture diverse attitudes within the broader population. This is recognized as a study limitation. | Page 11; line 354-361 |
| The manuscript relies on conventional content analysis without detailing how themes were derived, which may affect transparency and reproducibility. A more rigorous analytical framework, such as grounded theory or thematic analysis with inter-coder reliability checks, could strengthen the study’s validity. | Thank you for this important comment. We have revised the section and included more details on the procedure used for analysis, which was actually thematic in nature. | Page 3; Lines 120-132 |
| There is no mention of efforts to address potential biases in the FGDs, such as groupthink or interviewer influence, which could have affected the data. | Thank you for this important comment. We have included measures we used to control against potential bias during the FGDs. | Page 3; Lines 102-111 |
| Although the research mentions the gender distribution of participants, it does not delve deeply into how attitudes toward edible insects may vary by gender or other demographic factors such as age, education, or income levels. This omission weakens the ability to design tailored interventions. | Thank you for this important comment. We found some differences in knowledge and consumption among women/men; younger/older and lower/higher socio-economic levels. We have revised the results to include those different perspectives. | Page 8; Lines 230-233  Page 7; Lines 219-223  Page 8: Lines 240-255  Page 9; Lines 274-280  Page 12; Lines 361-368 |
| The paper briefly mentions seasonality as a factor limiting insect consumption but does not explore strategies to mitigate this challenge. For instance, addressing preservation methods or year-round farming could provide actionable insights. | Thank you for this important comment. We have included measures to address seasonality and supply challenges. | Page 12; Lines 389-391; 393-396 |
| The paper notes differences in acceptance between rural and urban areas but doesn't explore the underlying reasons for these differences in depth. Additional qualitative data could provide insights into cultural, economic, or educational factors that influence attitudes in these contrasting environments. | Thank you for this important comment. We agree with this suggestion. We did not find clear underlying reasons for differences in acceptability between rural and urban areas, except for the existence of edible insects in traditional diets. We have included some discussion on potential reasons for higher consumption in rural areas. | Page 10-11; Lines 312-321 |
| The suggestion to integrate traditional knowledge into food security policies is crucial but underdeveloped. More concrete examples of how traditional practices can inform modern interventions would make this recommendation more actionable. | This has been included in the manuscript as recommendations to be considered for national and county food policies- e.g. Kenya national nutrition action plan | Page 14; Lines 623-625 |
| The study repeats the need for promoting public awareness and improving availability of edible insects in multiple sections. Consolidating these ideas into a coherent discussion would improve clarity and avoid redundancy. | Thank you for this comment. We have harmonized the discussion on public awareness and improving availability to reduce redundancy as suggested. | Lines 476-477; 612-616 |
| The study does not compare its findings with other regions or countries where edible insect consumption is common. Including such comparisons could enrich the discussion and highlight best practices or transferable strategies. | The revised manuscript has included comparison with other studies in the discussion | Lines 432-449; 481-534; 537-545; 558-568 |
| While the study mentions curiosity and familiarity as factors influencing willingness to try insect-based products, it does not explore innovative ways to incorporate edible insects into widely accepted foods (e.g., snacks, flour). Addressing this could appeal to urban populations and younger demographics | Thank you for this important comment. We have included a discussion on curiosity and familiarity and incorporating edible insects in popular food items. | Page 11-12; Lines 332-370 |
